# Association of history of fracture with prehypertension and hypertension: a retrospective case–control study

**DOI:** 10.1186/s12891-015-0544-z

**Published:** 2015-04-12

**Authors:** Shuman Yang, Aimin Chen, Tianying Wu

**Affiliations:** Department of Environmental Health, Division of Epidemiology and Biostatistics, University of Cincinnati Medical Center, 3223 Eden Ave, Cincinnati, Ohio USA

**Keywords:** Fracture, Wrist fracture, High blood pressure, Prehypertension and hypertension

## Abstract

**Background:**

Hypertension is one of the most common encountered medical comorbidities after hip fracture. Whether fracture is a potential risk factor for hypertension remains poorly understood. The aim of our study was to examine the risk of prehypertension and hypertension in the participants with and without a history of fracture.

**Methods:**

We conducted a retrospective case–control study of 3,515 men and women aged between 20 and 85 years old from the National Health and Nutrition Examination Survey 2005–2006. History of fracture was collected via structured questionnaire. Multiple blood pressure readings (up to 4 times) were performed at interview, and an average of blood pressure readings were used to define prehypertension and hypertension.

**Results:**

Among 3,515 participants, 30% (n = 1074), 1.4% (n = 48) and 10% (n = 347) of them had a history of any, hip and wrist fracture, respectively. The positive association between history of any, hip and wrist fracture and prehypertension was similar to the association between history of any, hip and wrist fracture and hypertension in both unadjusted and adjusted model. In the unadjusted model, history of any, hip and wrist fracture was each associated with increased overall risk of prehypertension and hypertension (odds ratio [OR] = 1.61, 95% confidence interval [CI] = 1.38-1.89 for any fracture; OR = 3.57, 95% CI = 1.60-8.00 for hip fracture; and OR = 1.82, 95% CI = 1.41-2.36 for wrist fracture). However, in multivariable adjusted model, only the positive association between history of wrist fracture and overall risk of prehypertension and hypertension remained significant (OR = 1.48, 95% CI = 1.10-1.99).

**Conclusions:**

There was no overall independent association between history of fracture, and risk of prehypertension and hypertension. Although history of fracture overall may not directly cause hypertension, people with a history of wrist fracture can be potentially benefitted from hypertension control at the early stage.

**Electronic supplementary material:**

The online version of this article (doi:10.1186/s12891-015-0544-z) contains supplementary material, which is available to authorized users.

## Background

Fracture and hypertension represent major public health burden to health care system around the world because they are highly prevalent among general population. The residual lifetime risk of fracture from age 60 years was 44% for women and 25% for men [1]. The direct and indirect cost of fracture is enormous due to reduced mobility, increased requirements for hospitality and nursing home care [2,3]. Approximately 27% of men and 30% of women in the U.S. had hypertension between 1999 and 2000 [4]. The direct cost of hypertension treatment in U.S. between 1992 and 1993 was estimated at $3.8 billion [5]. The consequence of hypertension is not the disease itself, but its associated comorbidities including hemorrhagic stroke, ischemic brain lesions, silent brain infarcts, atherosclerosis, myocardial infarction and other cardiovascular diseases which are top killers worldwide [6-9].

Whether fracture is a potential risk factor for hypertension remains poorly understood. However, it has been suggested that hypertension is one of the most common encountered medical comorbidities after hip fracture [10]. Individuals with a fracture will generally have a limited mobility over a long period of time for post-fracture recovery. As sedentary lifestyle is a well-established risk factor for hypertension [11-14], history of fracture could be one of the causes for elevated blood pressure. In addition, human skeleton contains more than 90% of heavy metals from the environmental exposure which can be an endogenous source [15,16]. Fracture leads to bone loss [17,18], which causes long-term heavy metal leak from human skeleton. Following a fracture, increased circulating heavy metals could be another potential risk factor for hypertension [19].

On the basis of above statement, we therefore hypothesized that the risk of hypertension is higher in participants with a history of fracture than in those without the history of fracture. To test this hypothesis, we examined the risk of hypertension in participants with and without a history of fracture in a retrospective study of general population in U.S.

## Methods

### Study setting and subjects

**Figure 1:**
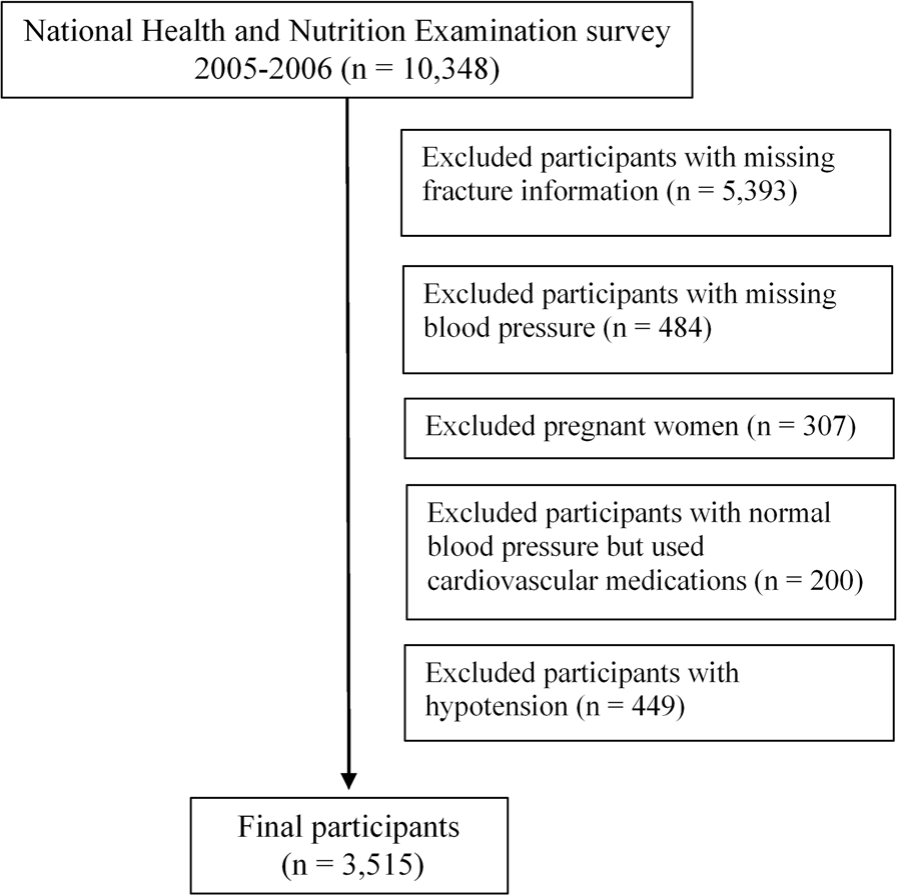
Flowchart of the participants excluded from the study. Use of cardiovascular medications (e.g., agents for hypertensive emergencies, angiotensin converting enzyme inhibitors, antiadrenergic agents [centrally acting], beta-adrenergic blocking agents, calcium channel blocking agents, diuretics and vasodilators) was ascertained during a one-month period prior to the date of interview.

The National Health and Nutrition Examination Survey (NHANES) is a continuous program examining the health and nutrition of a nationally representative population in the U.S. every year from 1990. The NHANES did not have individual follow-up, and almost all data were collected at interview. The retrospective case–control data of present study was a part of the NHANES, in which all data were collected between 2005 and 2006. Use of cardiovascular medications (e.g., agents for hypertensive emergencies, angiotensin converting enzyme inhibitors, antiadrenergic agents [centrally acting], beta-adrenergic blocking agents, calcium channel blocking agents, diuretics and vasodilators) was ascertained during a one-month period prior to the date of interview. As we did not know whether participants used certain cardiovascular medications for the treatment of hypertension or other cardiovascular diseases, and cardiovascular medications included blood pressure lowering components, we excluded all participants with normal blood pressure, but used cardiovascular medications (Figure 1). After excluding these participants, the chance of bias in normotensive diagnosis caused by blood pressure lowering medication in our study was very low. After excluding other ineligible participants (Figure 1), we finally included 3,515 men and women ages between 20 and 85 years old in our analysis. This investigation was exempt from review by the University of Cincinnati Institutional Review Board, but all participants provided written consent to participate in the NHANES.

### Demographic data collection

On the same day of blood pressure measurement, demographic data including age, gender, body mass index (BMI), history of diabetes, race and smoking status were collected by experienced interviewers. Smoking status included current, past and never smokers. Race was classified into five groups: Mexican American, Hispanic, non-Hispanic White, non-Hispanic Blacks and others. Physical activity, expressed in metabolic equivalent hours (MET-hours) per week, was estimated from the intensity and duration of the individual activities. One MET-hour is the energy expenditure for sitting quietly for one hour, which is approximately 3.5 ml of oxygen*kg body weight^−1^*min^−1^. Total fat intake was estimated from two 24-hour food recalls, in which detailed types and amounts of food and beverages were documented one day (from midnight to midnight) prior to the interview. The first 24-hour dietary recall interview was collected in-person at the Mobile Examination Center; the second interview was conducted by telephone 3–10 days later. The average fat intake estimated from two 24-hour recalls was used in our study.

### Fracture ascertainment

History of fracture including hip, wrist and other types of fracture was ascertained with structured questionnaire via direct interview, and other types of fracture included vertebral fracture and other fractures (except for hip, wrist and vertebral fracture). During the interview, data on type of fracture, age at fracture and circumstances of the fracture were collected. No validation was performed. All participants were free of fracture at the time of blood pressure measurement, and all blood pressure measurements were performed at least one year after fracture. Both traumatic and low-trauma fractures were analyzed in our study. Low trauma fracture was defined if it was due to a fall from standing height or less (i.e., tripped, slipped, fell out bed). Traumatic fracture was defined if the fracture was caused by a hard fall (i.e., falling off a ladder or step stool, down stair) or a car accident or other severe trauma. The fracture times for hip and wrist were also collected. We classified the history of fracture into three major groups: hip fracture, wrist fracture and any fracture.

### Hypotension, prehypertension and hypertension ascertainment

Each participant had a quiet rest for 5 minutes before blood pressure measurement. Blood pressure including systolic and diastolic blood pressure was measured by certified blood pressure examiners in the Mobile Examination Center. During the process, three consecutive blood pressure readings were obtained. The fourth reading was only made if one of previous blood pressure measurements was interrupted or incomplete. As the variation between multiple blood pressure readings for individuals was small, the mean of blood pressure readings (up to 4 times) was used to define hypotension, prehypertension and hypertension. According to the National Institute of Health (http://www.nhlbi.nih.gov/health/health-topics/topics/hyp/), we defined hypotension as blood pressure < 90/60 mm Hg. If participants did not have hypotension, and the blood pressure was ≥ 140/90 mm Hg, hypertension was defined (http://www.nhlbi.nih.gov/health/health-topics/topics/hbp/). If hypertension and hypotension were not defined above, and the blood pressure was ≥ 120/80 mm Hg, prehypertension was defined. The rest participants were normotensive.

### Statistical analysis

We compared the participants’ demographic characteristics in any-, hip-, and wrist-fracture group with that in non-fracture group. Then logistic regression model was used to analyze the association between participants’ demographic factors and risk of prehypertension and hypertension in non-fracture group.

In the logistic regression model, history of any, hip, or wrist fracture was exposure variable, and prehypertension and/or hypertension were outcomes. As compared with non-fracture group, each fracture group was analyzed with outcomes in three dichotomous logistic regression models separately. In the multivariate adjusted model, we included age, BMI, race, gender, alcohol intake, physical activity, total fat intake, history of diabetes and smoking status as covariates [20]. Although odds ratio (OR) may overestimate the actual risk when the prevalence of an outcome is over 10% [21], it has been suggested that OR is a more appropriate approach than relative risk (RR) [22]. To make a comprehensive analysis, we also presented RR and 95% confidence interval (CI) for a statistically significant relationship by using the method as mentioned previously [23]. We did not analyze the association between history of vertebral fracture and overall risk of prehypertension and hypertension separately, because such analysis showed an identical association as the overall association between history of any fracture and overall risk of prehypertension and hypertension in the multivariable adjusted model. All analyses were conducted with Statistical Analysis System (Version 9.3, SAS Institute Inc., Cary, NC) and R (Version 2.15.3, R Foundation for Statistical Computing).

## Results and discussion

### Results

#### Participants’ demographic characteristics stratified by any-, hip-, wrist- and non-fracture group

Among 3,515 participants, 30% (n = 1,074), 1.4% (n = 48) and 10% (n = 347) of them had a history of any, hip and wrist fracture, respectively (Table 1). Compared with non-fracture group, participants with history any fracture had lower proportion of female, Mexican American, Non-Hispanic Black, older age at the time of interview, higher total fat intake, and higher proportion of Non-Hispanic White, current and past smokers. Diagnosed prehypertension and hypertension were higher among participants with history of any fracture than those without history of fracture. Compared with non-fracture group, participants’ demographic characteristics in hip- and wrist-fracture group were comparable to that in any-fracture group.

**Table 1:** **Participants’ demographic characteristics stratified by any**-**, hip**-**, wrist**- **and non**-**fracture group** (**n** = **3,515)**

	**Any-** **fracture group**	**Hip-** **fracture group**	**Wrist-** **fracture group**	**Non-** **fracture group**
n	1074	48	347	2441
Female (n, %)	467 (43.5%)**	21 (43.8%)	148 (42.7%)	1204 (49.3%)
Age at interview (years)	54.4 (17.9)**	66.6 (18.6)**	52.3 (18.8)**	47.7 (17.9)
Age of first fracture (years)^±^	26 (15, 45)	53 (25, 74)	17 (11, 40)	---
Body mass index (kg/m^2^)	28.7 (6.3)	27.9 (8.5)	28.3 (5.9)	28.8 (6.8)
Physical activity (MET-hours/week)^±^	13.8 (5.1, 35.5)	11.7 (5.1, 24.9)	14.2 (5.5, 37.8)	14.6 (5.3, 33.1)
Total fat intake (g/day)^±^	73.4 (52.1, 101.1)*	72.6 (48.8, 93.3)	74.5 (50.9, 108.5)	70.5 (50.1, 97.9)
Alcohol intake ≥ 3 drinks/day (n, %)	269 (25.1%)	8 (16.7%)	103 (29.7%)*	577 (23.6%)
History of diabetes (n, %)	110 (10.2%)	5 (10.4%)	34 (9.8%)	242 (9.9%)
**Race**				
Mexican American (n, %)	140 (13.0%)**	5 (10.4%)	44 (12.7%)**	547 (22.4%)
Hispanic (n, %)	13 (1.2%)	1 (2.1%)	6 (1.7%)	91 (3.7%)
Non-Hispanic White (n, %)	726 (67.6%)**	33 (68.8%)	252 (72.6%)**	1050 (43.0%)
Non-Hispanic Black (n, %)	163 (15.2%)**	6 (12.5%)	38 (11.0%)**	647 (26.5%)
Others (n, %)	32 (3.0%)	3 (6.3%)	7 (2.0%)	106 (4.3%)
**Smokers**				
Current (n, %)	283 (26.4%)**	7 (14.6%)	93 (26.8%)**	503 (20.6%)
Past (n, %)	269 (25.1%)**	15 (31.3%)	91 (26.2%)**	477 (19.5%)
Prehypertension (n, %)	504 (46.9%)**	26 (54.2%)**	164 (47.3%)**	975 (39.9%)
Hypertension (n, %)	275 (25.6%)**	15 (31.3%)**	96 (27.7%)**	541 (22.2%)

Values with normal distribution are shown in means (standard deviation), unless otherwise specified. ^±^Values with skew distribution are shown in medians (inter-quartile range).

Statistical significance against non-fracture group: *P < 0.05; **P < 0.01.

MET = Metabolic equivalent.

### Participants’ demographic factors and risk of prehypertension and hypertension among non-fracture group

The patterns of demographic factors associated with risk prehypertension and hypertension were comparable. In the bivariate analysis, being male gender, increase in age, higher BMI, history of diabetes and past smokers were associated with increased risk of prehypertension and hypertension (Table 2). Mexican American, Hispanic and other races had lower risk of prehypertension and hypertension than Non-Hispanic White. However, risk of prehypertension and hypertension was higher in Non-Hispanic Black than in Non-Hispanic White.

**Table 2:** **Association between participants’ demographic factors and risk of prehypertension and hypertension among non**-**fracture group: bivariate analysis (n = 2,441)**

**Variables**	**Per unit or comparison**	**Prehypertension**	**Hypertension**	**Prehypertension and hypertension**
Gender	Male vs. female	**2.22 (1.85, 2.67)**	**1.58 (1.27, 1.95)**	**1.96 (1.66, 2.32)**
Age (years)	Per SD increase^±^	**2.35 (2.09, 2.64)**	**6.17 (5.15, 7.39)**	**2.98 (2.67, 3.32)**
Body mass index (kg/m^2^)	≥35 vs. < 25	**2.98 (2.20, 4.03)**	**2.71 (1.88, 3.90)**	**2.89 (2.18, 3.83)**
Physical activity (MET-hours/week)	Quartile 4 vs. 1	0.93 (0.67, 1.28)	0.70 (0.47, 1.04)	0.84 (0.63, 1.13)
Total fat intake (g/day)	Quartile 4 vs. 1	1.05 (0.81, 1.36)	**0.55 (0.40, 0.75)**	0.84 (0.66, 1.07)
Alcohol intake (drinks/day)	≥3 vs. 1-2	1.06 (0.84, 1.33)	0.74 (0.55, 1.01)	0.96 (0.77, 1.18)
History of diabetes	Yes vs. no	**4.38 (2.85, 6.74)**	**7.62 (4.91, 11.83)**	**5.48 (3.64, 8.24)**
Race	Mexican American vs. Non-Hispanic White	**0.67 (0.54, 0.85)**	**0.46 (0.34, 0.61)**	**0.59 (0.48, 0.73)**
	Hispanic vs. Non-Hispanic White	**0.61 (0.38, 0.98)**	**0.50 (0.28, 0.92)**	**0.57 (0.37, 0.88)**
	Non-Hispanic Black vs. Non-Hispanic White	**1.37 (1.09, 1.73)**	**1.49 (1.15, 1.94)**	**1.41 (1.14, 1.75)**
	Others vs. Non-Hispanic White	**0.54 (0.34, 0.84)**	**0.43 (0.24, 0.77)**	**0.50 (0.33, 0.74)**
Smokers	Current vs. never	1.02 (0.65, 1.59)	0.87 (0.52, 1.47)	0.96 (0.64, 1.44)
	Past vs. never	**2.01 (1.27, 3.18)**	**1.85 (1.09, 3.14)**	**1.95 (1.29, 2.95)**

Values are odds ratio (95% confidence interval). ^±^18 years. Bold-faced values indicate statistical significance at *P* < 0.05.

### History of fracture and hypertension

The positive association between history of any, hip and wrist fracture and prehypertension was similar to the association between history of any, hip and wrist fracture and hypertension in both unadjusted and adjusted model (Table 3). In the unadjusted model, history of any, hip and wrist fracture was each associated with increased overall risk of prehypertension and hypertension (OR = 1.61, 95% CI = 1.38-1.89 for any fracture; OR = 3.57, 95% CI = 1.60-8.00 for hip fracture; and OR = 1.82, 95% CI = 1.41-2.36 for wrist fracture). However, in multivariable adjusted model, only the positive association between history of wrist fracture and overall risk of prehypertension and hypertension remained significant (OR = 1.48, 95% CI = 1.10-1.99). When we present the data in RR, the association between history of wrist fracture and overall risk of prehypertension and hypertension was still significant (RR = 1.08, 95% CI = 1.03-1.14).

**Table 3:** **Association between history of any, hip and wrist fracture, and risk of prehypertension and hypertension: multiple logistic regression models**

**Models**	**Type of fracture**	**Prehypertension**	**Hypertension**	**Prehypertension and hypertension**
Unadjusted	Any fracture	**1.62 (1.37, 1.92)**	**1.59 (1.31, 1.94)**	**1.61 (1.38, 1.89)**
	Hip fracture	**3.52 (1.52, 8.16)**	**3.66 (1.48, 9.04)**	**3.57 (1.60, 8.00)**
	Wrist fracture	**1.79 (1.36, 2.36)**	**1.89 (1.39, 2.57)**	**1.82 (1.41, 2.36)**
Adjusted for multiple risk factors*	Any fracture	1.19 (0.98, 1.45)	0.96 (0.73, 1.27)	1.13 (0.94, 1.36)
	Hip fracture	1.83 (0.73, 4.63)	0.64 (0.20, 2.07)	1.37 (0.55, 3.45)
	Wrist fracture	**1.49 (1.09, 2.03)**	1.53 (0.99, 2.37)	**1.48 (1.10, 1.99)**

Values are odds ratio (95% confidence interval). Bold-faced values indicate statistical significance at *P* < 0.05. *Risk factors included age (continuous), body mass index (<25, ≥25 and <30, ≥30 and <35, and ≥35 kg/m^2^), race (Mexican American, Hispanic, Non-Hispanic White, Non-Hispanic Black, and others), gender (male and female), alcohol intake (1–2, and 3+ drinks/day), physical activity (in quartiles), total fat intake (in quartiles), history of diabetes (yes/no), smokers (current, past, and never smokers).

Further analysis of the association between history of wrist fracture and overall risk of prehypertension and hypertension suggested that the relationship participants with female gender, 2 or more times of history of wrist fracture, less than 20 years between age of first wrist fracture and age of blood pressure measurement, and low trauma wrist fracture had higher overall risk of prehypertension and hypertension than those without the history of fracture (Additional file 1: Table S1). Further, the associations between history of wrist fracture and overall risk of prehypertension and hypertension in different age groups of first wrist fracture were comparable.

As we found only wrist fracture was associated with increased risk of prehypertension and hypertension, we further examined the participants’ demographic characteristics stratified by wrist-fracture and other-fracture group (Additional file 1: Table S2). We found that participants with wrist fracture were much younger at interview and at the time of first fracture, had higher alcohol intake, were more likely to be non-Hispanic White, and were less likely to be non-Hispanic Black than those with other fractures (Additional file 1: Table S2).

## Discussion

Although hypertension has been suggested to be an independent risk factor for fractures [24-26], whether fracture play a role in the development of hypertension is still unclear. In this large-scale retrospective study of the U.S. general population, we have demonstrated that there was no overall independent association between history of fracture, and risk of prehypertension and hypertension. Interestingly, history of wrist fracture appeared to have an independent association with high blood pressure. Although history of fracture overall may not directly cause hypertension, our results suggested that history of wrist fracture may have values for hypertension control at the early stage.

We found that history of wrist fracture, but not other fractures, was associated with increased overall risk of prehypertension and hypertension. The reason for this is still unclear. However, as shown in Additional file 1: Table S2, the participants with a history of wrist fracture had greater alcohol intake than those with a history of other fractures. Thus, elevated blood pressure may be attributed to the greater alcohol intake [27] which is also shown to be associated with poorer cardiovascular self-care behaviors [28]. In addition, similar to other fractures, wrist fracture can cause limited mobility, psychological distress, pain and certain level of heavy metal leaks, which are potential risk factors for prehypertension and hypertension [16,17,29]. Certainly, as the overall independent association between history of fracture and overall risk of prehypertension and hypertension is lacking, we cannot exclude the possibility that the positive relationship between wrist fracture and overall risk of prehypertension and hypertension was due to chance or other unknown mediated factors.

The findings of the current study may have clinical implications at population level, as both wrist fracture and hypertension are highly prevalent among general population. Wrist fracture generally occurs at an earlier age. Although there are many risk factors cause hypertension, an earlier marker for assessing the risk of hypertension is still lacking. In our study, the relationship between history of wrist fracture and high blood pressure may not be direct. However, history of wrist fracture may reflect some important risk factors of hypertension because the proportion of hypertension was higher in participants with a history of wrist fracture than in those without the history. Thus, history of wrist fracture can be regarded as an early and surrogate marker for important risk factors for hypertension. This has raised the awareness that patients with a history of wrist fracture may potentially benefit from the regular blood pressure control at the early stage.

The primary limitation of present study is that we did not know whether participants had hypertension at the time of wrist fracture. However, majority of participants with a history of wrist fracture unlikely had high blood pressure at the time of fracture, because more than 50% of them were less than 20 years old at the time of fracture (see Additional file 1: Table S1). All fractures were self-reported, and no validation was conducted. Moreover, we cannot exclude the possibility of recall bias due to the retrospective study design. All of these factors could lead to misclassification between fracture and non-fracture groups. In addition, the independent positive association between history of wrist fracture and overall risk of prehypertension and hypertension may be biased, because the duration between age of wrist fracture and age of blood pressure measurement was really long. Lastly, the results of our study may be influenced by some unknown confounders due to the study design. Therefore, the finding between history of wrist fracture and overall risk of prehypertension and hypertension can only be interpreted as an association, but not a causation.

The major advantage of present study is that we included a large sample size and high proportion of hypertension cases. In addition, hypertension cases in our study were defined from multiple blood pressure readings. Thus, the reliability of the hypertension ascertainment is high.

## Conclusions

History of fracture is not independently associated with increased risk of prehypertension and hypertension in general U.S. population. Wrist fracture appeared to be stronger than other fractures in relation to hypertension. Due to retrospective study design, our study warrants further validation and confirmation in a prospective setting.

## Additional file

Additional file 1: Table S1. Sensitivity analysis of association between history of wrist fracture and overall risk of prehypertension and hypertension. **Table S2.** Participants’ demographic characteristics stratified by wrist- and other- fracture group (n =1,074).

## Abbreviations

BMI: Body mass index
CI: Confidence interval
MET: Metabolic equivalent
NHANES: National Health and Nutrition Examination Survey
OR: Odds ratio
RR: Relative risk
