# Supplementary material for: Association of history of fracture with prehypertension and hypertension: a retrospective case–control study
Source: BMC Musculoskelet Disord. 2015 Apr 12;16:86. doi: 10.1186/s12891-015-0544-z (PMC4410583; doi:10.1186/s12891-015-0544-z)
Supplement: Additional file 1: Table S1. — Sensitivity analysis of association between history of wrist fracture and overall risk of prehypertension and hypertension. Table S2. Participants’ demographic characteristics stratified by wrist- and other- fracture group (n =1,074). [file 12891_2015_544_MOESM1_ESM.docx]

**Table S1. Sensitivity analysis of association between history of wrist fracture and overall risk of prehypertension and hypertension**

| Subgroups | No. of wrist fracture | Prehypertension and hypertension |
| --- | --- | --- |
| **Gender**  Men  Women | 199  148 | 1.29 (0.89, 1.87)  1.68 (0.99, 2.83) |
| **Times of wrist fracture**  1 time only  2+ times | 288  59 | 1.34 (0.97, 1.84)  **2.38 (1.18, 4.81)** |
| **Age of first wrist fracture**  < 20 years  21-39 years  40+ years  Missing information | 190  58  96  3 | 1.31 (0.92, 1.89)  1.80 (0.91, 3.55)  1.74 (0.86, 3.50)  --- |
| **Duration between age of the first wrist fracture and age of blood pressure measurement**  ≤ 20 years  21+ years  Missing information | 162  182  3 | **1.95 (1.28, 2.96)**  1.11 (0.75, 1.64)  --- |
| **Cause of the first wrist fracture**  Low trauma  High trauma  Missing information | 46  22  279 | **2.13 (1.16, 3.92)**  1.46 (0.63, 3.37)  --- |

Values are odds ratio (95% confidence interval). *Odds ratios were adjusted for age (continuous), body mass index (<25, ≥25 and <30, ≥30 and <35, and ≥35 kg/m^2^), race (Mexican American, Hispanic, Non-Hispanic White, Non-Hispanic Black, and others), gender (male and female), alcohol intake (1-2, and 3+ drinks/day), physical activity (in quartiles), total fat intake (in quartiles), history of diabetes (yes/no), smokers (current, past, and never smokers).

**Table S2. Participants’ demographic characteristics stratified by wrist- and other- fracture group (n =1,074)**

|  | Wrist-fracture group | Other-fracture group |
| --- | --- | --- |
| n | 347 | 727 |
| Female (n, %) | 148 (42.7%) | 319 (43.9%) |
| Age at interview (years) | **52.3 (18.8)**** | **55.4 (17.5)** |
| Age of first fracture (years) ^±^ | **17 (11, 40)**** | **34 (24, 50)** |
| Body mass index (kg/m^2^) | 28.3 (5.9) | 29.0 (6.5) |
| Physical activity (MET-hours/week)^±^ | 14.2 (5.5, 37.8) | 13.7 (4.9, 35.0) |
| Total fat intake (g/day) ^±^ | 74.5 (50.9, 108.5) | 73.0 (52.6, 96.9) |
| Alcohol intake ≥ 3 drinks/day (n, %) | **103 (29.7%)*** | **166 (22.8%)** |
| History of diabetes (n, %) | 34 (9.8%) | 76 (10.5%) |
| **Race** |  |  |
| Mexican American (n, %) | 44 (12.7%) | 96 (13.2%) |
| Hispanic (n, %) | 6 (1.7%) | 7 (1.0%) |
| Non-Hispanic White (n, %) | **252 (72.6%)*** | **474 (65.2%)** |
| Non-Hispanic Black (n, %) | **38 (11.0%)**** | **125 (17.2%)** |
| Others (n, %) | 7 (2.0%) | 25 (3.4%) |
| **Smokers** |  |  |
| Current (n, %) | 93 (26.8%) | 190 (26.1%) |
| Past (n, %) | 91 (26.2%) | 178 (24.5%) |

Values with normal distribution are shown in means (standard deviation), unless otherwise specified. ^±^Values with skew distribution are shown in medians (inter-quartile range).

Statistical significance against other-fracture group: **P* < 0.05; ***P* < 0.01.

MET = Metabolic equivalent.
